# Magnesium Depletion Score and Metabolic Syndrome in US Adults: Analysis of NHANES 2003 to 2018

**DOI:** 10.1210/clinem/dgae075

**Published:** 2024-02-14

**Authors:** Xiaohao Wang, Zhaohao Zeng, Xinyu Wang, Pengfei Zhao, Lijiao Xiong, Tingfeng Liao, Runzhu Yuan, Shu Yang, Lin Kang, Zhen Liang

**Affiliations:** Department of Geriatrics, The First Affiliated Hospital, School of Medicine, Southern University of Science and Technology (Shenzhen People's Hospital), Shenzhen 518055, China; Department of Geriatrics, Shenzhen People's Hospital (The First Affiliated Hospital, Southern University of Science and Technology; The Second Clinical Medical College, Jinan University), Shenzhen 518055, China; Shenzhen Clinical Research Centre for Geriatrics, Shenzhen People's Hospital, Shenzhen 518055, China; Department of Neurology, Shenzhen People's Hospital (The Second Clinical Medical College, Jinan University, The First Affiliated Hospital, Southern University of Science and Technology), Shenzhen 518055, China; Department of Nephrology, The First People's Hospital of Yunnan Province, The Affiliated Hospital of Kunming University of Science and Technology, Kunming, Yunnan 650034, China; Department of Geriatrics, The First Affiliated Hospital, School of Medicine, Southern University of Science and Technology (Shenzhen People's Hospital), Shenzhen 518055, China; Department of Geriatrics, Shenzhen People's Hospital (The First Affiliated Hospital, Southern University of Science and Technology; The Second Clinical Medical College, Jinan University), Shenzhen 518055, China; Shenzhen Clinical Research Centre for Geriatrics, Shenzhen People's Hospital, Shenzhen 518055, China; Department of Geriatrics, The First Affiliated Hospital, School of Medicine, Southern University of Science and Technology (Shenzhen People's Hospital), Shenzhen 518055, China; Department of Geriatrics, Shenzhen People's Hospital (The First Affiliated Hospital, Southern University of Science and Technology; The Second Clinical Medical College, Jinan University), Shenzhen 518055, China; Shenzhen Clinical Research Centre for Geriatrics, Shenzhen People's Hospital, Shenzhen 518055, China; Department of Geriatrics, The First Affiliated Hospital, School of Medicine, Southern University of Science and Technology (Shenzhen People's Hospital), Shenzhen 518055, China; Department of Geriatrics, Shenzhen People's Hospital (The First Affiliated Hospital, Southern University of Science and Technology; The Second Clinical Medical College, Jinan University), Shenzhen 518055, China; Shenzhen Clinical Research Centre for Geriatrics, Shenzhen People's Hospital, Shenzhen 518055, China; Department of Geriatrics, The First Affiliated Hospital, School of Medicine, Southern University of Science and Technology (Shenzhen People's Hospital), Shenzhen 518055, China; Department of Geriatrics, Shenzhen People's Hospital (The First Affiliated Hospital, Southern University of Science and Technology; The Second Clinical Medical College, Jinan University), Shenzhen 518055, China; Shenzhen Clinical Research Centre for Geriatrics, Shenzhen People's Hospital, Shenzhen 518055, China; Department of Geriatrics, The First Affiliated Hospital, School of Medicine, Southern University of Science and Technology (Shenzhen People's Hospital), Shenzhen 518055, China; Department of Geriatrics, Shenzhen People's Hospital (The First Affiliated Hospital, Southern University of Science and Technology; The Second Clinical Medical College, Jinan University), Shenzhen 518055, China; Shenzhen Clinical Research Centre for Geriatrics, Shenzhen People's Hospital, Shenzhen 518055, China; Department of Geriatrics, The First Affiliated Hospital, School of Medicine, Southern University of Science and Technology (Shenzhen People's Hospital), Shenzhen 518055, China; Department of Geriatrics, Shenzhen People's Hospital (The First Affiliated Hospital, Southern University of Science and Technology; The Second Clinical Medical College, Jinan University), Shenzhen 518055, China; Shenzhen Clinical Research Centre for Geriatrics, Shenzhen People's Hospital, Shenzhen 518055, China; Department of Geriatrics, The First Affiliated Hospital, School of Medicine, Southern University of Science and Technology (Shenzhen People's Hospital), Shenzhen 518055, China; Department of Geriatrics, Shenzhen People's Hospital (The First Affiliated Hospital, Southern University of Science and Technology; The Second Clinical Medical College, Jinan University), Shenzhen 518055, China; Shenzhen Clinical Research Centre for Geriatrics, Shenzhen People's Hospital, Shenzhen 518055, China

**Keywords:** magnesium, magnesium depletion score (MDS), metabolic syndrome (MetS), NHANES, nutritional epidemiology

## Abstract

**Context:**

The association between magnesium status and metabolic syndrome (MetS) remains unclear.

**Objective:**

This study aimed to examine the relationship between kidney reabsorption-related magnesium depletion score (MDS) and MetS among US adults.

**Methods:**

We analyzed data from 15 565 adults participating in the National Health and Nutrition Examination Survey (NHANES) 2003 to 2018. MetS was defined according to the National Cholesterol Education Program's Adult Treatment Panel III report. The MDS is a scoring system developed to predict the status of magnesium deficiency that fully considers the pathophysiological factors influencing the kidneys' reabsorption capability. Weighted univariate and multivariable logistic regression were used to assess the association between MDS and MetS. Restricted cubic spline (RCS) analysis was conducted to characterize dose-response relationships. Stratified analyses by sociodemographic and lifestyle factors were also performed.

**Results:**

In both univariate and multivariable analyses, higher MDS was significantly associated with increased odds of MetS. Each unit increase in MDS was associated with approximately a 30% higher risk for MetS, even after adjusting for confounding factors (odds ratio 1.31; 95% CI, 1.17-1.45). RCS graphs depicted a linear dose-response relationship across the MDS range. This positive correlation remained consistent across various population subgroups and exhibited no significant interaction by age, sex, race, adiposity, smoking status, or alcohol consumption.

**Conclusion:**

Higher urinary magnesium loss as quantified by MDS may be an independent linear risk factor for MetS in US adults, irrespective of sociodemographic and behavioral factors. Optimizing magnesium nutritional status could potentially confer benefits to patients with MetS.

Metabolic syndrome (MetS) is a common metabolic disorder caused by the increasing prevalence of obesity, and currently, this disorder is defined in various ways. In the 1980s, Reaven clustered the risk factors for diabetes and cardiovascular diseases together and called it “syndrome X,” which includes insulin resistance, hypertension, hyperglycemia, low high-density lipoprotein cholesterol (HDL-c), and high triglycerides (TGs) (1). Insulin resistance is defined clinically as the inability of a known quantity of exogenous or endogenous insulin to increase glucose uptake and utilization in an individual as much as it does in a normal population (2). In 1992, after central obesity was added as a core component of the definition and entity, it was renamed insulin resistance syndrome (IRS) (3). In 1998, The World Health Organization uniformly named it “metabolic syndrome” (MetS) and proposed that insulin resistance is the core of the pathophysiology of MetS (4). In the following years, the definitions of MetS revised according to the racial conditions of other countries also appeared, among which the more influential ones include the definition of the National Cholesterol Education Program Adult Treatment Panel III Criteria (NCEP-ATP III) (5) and the definition of the International Diabetes Federation. According to the NCEP ATP III definition, MetS is diagnosed as the presence of at least 3 of the following 5 characteristics: high waist circumference, high blood pressure, elevated blood sugar level, increased TGs, and low HDL-c (5). MetS is important because of its association with an increasing prevalence of diabetes and a higher risk of cardiovascular events such as heart disease and stroke, which have become a major public health challenge.

Magnesium is a vital mineral in the human body, serving as an essential cofactor for hundreds of enzymatic reactions. These include energy metabolism, protein and nucleic acid synthesis, and the secretion and action of insulin (6, 7). It plays a crucial role in maintaining normal nerve and muscle function, supporting a healthy immune system, and ensuring a steady heartbeat. Despite its importance, magnesium deficiency is a widespread and often overlooked public health issue. More than half of the US population fails to meet the recommended dietary allowance for magnesium intake, leading to a high estimated prevalence (∼15%) of magnesium deficiency (8). Studies have found that hypomagnesemia is associated with elevated risk and adverse prognosis in patients with diabetes, stroke, and coronary artery disease. Increasing magnesium intake can reduce the risk of diabetes, stroke and hypertension (9, 10).

Although serum magnesium is frequently used in clinical settings to assess magnesium deficiency (11), it may not accurately reflect the body's overall magnesium status. This is largely due to the kidneys’ role in absorbing more than 80% of plasma magnesium, a process vital for maintaining magnesium homeostasis. In light of this, Fan et al (12) developed the magnesium depletion score (MDS), a composite score that aggregates 4 established risk factors and also takes into account the pathophysiological factors influencing the kidneys’ reabsorption capability. A higher MDS indicates a more severe state of magnesium deficiency. However, to our knowledge, the association between the MDS and MetS has not been studied. It may therefore be helpful to further explore the relationship between MDS and MetS to develop future prevention or treatment methods for this disease. We investigated the association between MDS and MetS by examining 80 312 participants from the NHANES from 2003 to 2018.

## Materials and Methods

### Study Population and Design

**Figure 1.**
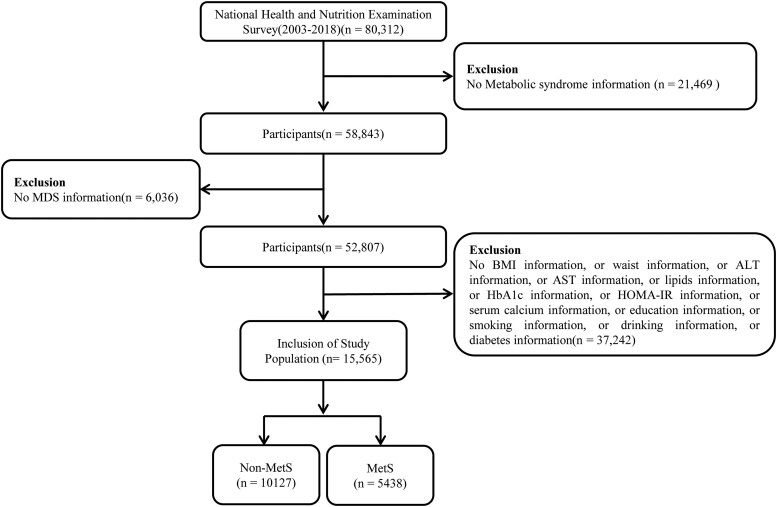
Flowchart of participant selection from National Health and Nutrition Examination Survey 2003 to 2018.

Data for this study were obtained from the National Health and Nutrition Examination Survey website (https://www.cdc.gov/nchs/nhanes/index.htm). In this study, 9 cycles of NHANES data (2003-2018) were collected by the Centers for Disease Control and Prevention. NHANES is a multistage, stratified, large, nationally representative study of the US population that provides detailed information about the study design, interviews, demographics, etc (13-15). Among the sample of 80 312 adults, those who did not provide MDS information or other variables were excluded (Fig. 1). Protocol approval was granted by the National Center for Health Statistics’ ethical review board, and informed consent forms were signed by participants.

### Calculation of the Magnesium Depletion Score

The MDS was calculated to evaluate total-body magnesium status. The MDS was calculated as the sum of the following 4 scores: (1) current use of diuretics was scored 1 point; (2) current use of proton pump inhibitor (PPI) was scored 1 point; (3) an estimated glomerular filtration rate (eGFR) between 60 mL/min/1.73 m^2^ and 90 mL/min/1.73 m^2^ was scored 1 point, while an eGFR less than 60 mL/min/1.73 m^2^ was scored 2 points; and (4) heavy drinking (>1 drink/d for women and >2 drinks/d for men) was scored 1 point (12). The eGFR was calculated using the Chronic Kidney Disease Epidemiology Collaboration formula (16). According to the Food Patterns Equivalents Database, alcoholic drinks include all types of beers, wines, distilled spirits (such as brandy, gin, rum, vodka, and whiskey), cordials, and liqueurs. One drink was defined as the amount of alcoholic beverage containing 0.6 fluid ounces or 14 g of ethanol. MDS was categorized into 6 groups: MDS = 0, MDS = 1, MDS = 2, MDS = 3, MDS = 4, and MDS = 5.

### Diagnosis of Metabolic Syndrome

MetS was defined according to the NCEP-ATP III report (5). Participants were diagnosed with MetS if they met at least 3 of the following 5 criteria: (1) central obesity: waist circumference greater than or equal to 102 cm in men, or greater than or equal to 88 cm in women; (2) hypertriglyceridemia: serum TGs greater than or equal to 150 mg/dL; (3) low HDL-c: serum HDL-c less than 40 mg/dL in men and less than 50 mg/dL in women; (4) hypertension: systolic blood pressure (SBP) greater than or equal to 130 mm Hg, or diastolic blood pressure (DBP) greater than or equal to 85 mm Hg, or receiving antihypertensive treatment; (5) hyperglycemia: fasting glucose greater than or equal to 100 mg/dL, or receiving antihyperglycemic treatments. Data on waist circumference, body weight, and height were collected using standard procedures during physical examinations. SBP and DBP were calculated as the arithmetic averages of repeated measures (up to 4 times) for each participant. TGs and HDL-c were measured in serum, while fasting glucose was measured in plasma.

### Covariates Assessment

NHANES collects some sociodemographic information through structured data. The main covariates in this study were demographic characteristics and chronic comorbidities. Demographic characteristics, including age, sex, race, and education level, were reported by interviewees. Measurements of waist size, weight, and height were taken by well-trained health technologists following the anthropometry procedure manual. Body mass index (BMI) was calculated as weight (kg) divided by height squared (m^2^). Currently used clinical criteria and guidelines issued by the International Diabetes Association were used to diagnose diabetes, with diabetes classified by either fasting blood glucose greater than or equal to 7.0 mmol/L in laboratory tests, blood glucose greater than 11.1 mmol/L in the oral glucose tolerance test experiment, those taking diabetes medications, those diagnosed with diabetes by their doctors during the survey, and those who self-reported. Hypertension was defined as a mean SBP greater than or equal to 140 mm Hg or DBP greater than or equal to 90 mm Hg or current use of antihypertensive medication as per self-reported questionnaire. Other chronic comorbidities such as hyperlipidemia were identified either through a doctor's diagnosis or a self-reported questionnaire.

### Statistical Analyses

For all individuals in the present study, descriptive analyses were performed on the characteristics of each participant. Statistical analysis was conducted using R open-source software version 4.3.1 to analyze the data for this study. Data extraction and analyses were performed with the use of the “nhanesR” package of R software(R Core Team; 2023. R: A Language and Environment for Statistical Computing. R Foundation for Statistical Computing, https://www.R-project.org/). For continuous variables, the mean and SD were reported, while for categorical variables, percentages were reported. For continuous variables, the baseline characteristics were analyzed using a linear regression model, and for categorical variables, they were analyzed using a chi-square test. Weighted univariate and multivariable logistic regression were used to assess the association between MDS and MetS. The formulas for multivariable logistic regression were: log(p/(1 − p)) = β0 + β1X1 + β2X2 + … + βnXn (where p = probability of MetS, β0 = intercept, β1 to βn = regression coefficients for each predictor variable, X1 to Xn = predictor variables (age, sex, race, etc). Four models were created: a crude model with no adjustments for confounding factors; model 1, adjusted for age, sex, race, and education level; model 2, adjusted for the variables in model 1 plus BMI, waist, alanine transaminase (ALT), aspartate transaminase (AST), TGs, total cholesterol (TC), HDL-c, low-density lipoprotein cholesterol (LDL-c), homeostatic model assessment for insulin resistance (HOMA-IR), glycated hemoglobin A_1c_ (HbA_1c_), corrected calcium (Ca), smoking, and drinking; and model 3, adjusted for the variables in model 2 plus hyperlipidemia, diabetes, and hypertension. Restricted cubic spline (RCS) analysis was conducted to characterize dose-response relationships. Stratified analyses by sociodemographic and lifestyle factors were also performed. We considered all survey sampling weights when analyzing the data, which were considered statistically significant if *P* was less than .05.

## Results

### Baseline Characteristics of Study Participants by Metabolic Syndrome

Between 2003 and 2018, a total of 80 312 participants were involved in NHANES (see Fig. 1). Participants who did not provide relevant information for the calculation of MetS, MDS, or other variables were excluded, resulting in a final sample of 15 565 participants for statistical analysis. Of these, 10 127 did not have MetS, while 5438 did. In Table 1, we show the general characteristics of the study population based on whether they had MetS. Compared to individuals without MetS, those with MetS were found to be more likely older, of non-Hispanic white ethnicity, smokers, nondrinkers, and with a lower education level. They also exhibited higher BMI, waist circumference, ALT, AST, TGs, TC, LDL-c, and MDS, but lower eGFR. Furthermore, individuals with MetS demonstrated more severe insulin resistance, higher levels of HbA_1c_, and were more likely to have a history of hyperlipidemia, hypertension, and diabetes. MDS was categorized into 6 levels from 0 to 5. The proportion of participants with MDS = 0 was lower in the MetS group compared to the non-MetS group (32.48% vs 49.41%), while the proportions for MDS greater than or equal to 2 were all higher in the MetS group (all *P* < .05).

**Table 1.** Baseline characteristics of participants with or without metabolic syndrome

Characters	Total	Non–metabolic syndrome	Metabolic syndrome	*P*
Age, y	47.3 ± 0.3	43.7 ± 0.3	54.7 ± 0.3	**<.0001**
Sex				.06
Male	7823 (49.78%)	5278 (50.54%)	2545 (48.20%)	
Female	7744 (50.22%)	4851 (49.46%)	2893 (51.80%)	
Race				**<.0001**
Non-Hispanic White	7029 (69.83%)	4411 (68.37%)	2618 (72.85%)	
Non-Hispanic Black	3115 (10.15%)	2108 (10.66%)	1007 (9.09%)	
Mexican American	2544 (8.24%)	1613 (8.35%)	931 (8.03%)	
Other race	2879 (11.78%)	1997 (12.62%)	882 (10.03%)	
Education				**<.0001**
> High school	8029 (59.90%)	5539 (62.99%)	2490 (53.50%)	
High school	5955 (34.89%)	3704 (32.42%)	2251 (39.99%)	
< High school	1583 (5.21%)	886 (4.59%)	697 (6.51%)	
BMI	28.83 ± 0.09	26.70 ± 0.09	33.25 ± 0.15	**<.0001**
Waist, cm	98.88 ± 0.23	92.94 ± 0.21	111.18 ± 0.33	**<.0001**
MDS	0.83 ± 0.01	0.67 ± 0.01	1.16 ± 0.02	**<.0001**
MDS				**<.0001**
0	7135 (44.23%)	5366 (49.91%)	1769 (32.48%)	
1	5057 (35.79%)	3352 (36.76%)	1705 (33.78%)	
2	2292 (14.23%)	1087 (10.65%)	1205 (21.65%)	
3	885 (4.67%)	276 (2.25%)	609 (9.66%)	
4	192 (1.05%)	45 (0.41%)	147 (2.39%)	
5	6 (0.03%)	3 (0.02%)	3 (0.03%)	
ALT, U/L	25.27 ± 0.17	23.77 ± 0.18	28.37 ± 0.34	**<.0001**
AST, U/L	25.07 ± 0.16	24.60 ± 0.16	26.04 ± 0.29	**<.0001**
TGs, mmol/L	1.35 ± 0.01	1.09 ± 0.01	1.90 ± 0.02	**<.0001**
TC, mmol/L	5.01 ± 0.01	4.99 ± 0.01	5.06 ± 0.02	**.002**
HDL-c, mmol/L	1.41 ± 0.01	1.51 ± 0.01	1.21 ± 0.01	**<.0001**
LDL-c, mmol/L	2.96 ± 0.01	2.94 ± 0.01	2.99 ± 0.02	**.02**
eGFR, mL/min/1.73 m^2^	94.86 ± 0.34	98.22 ± 0.36	87.90 ± 0.48	**<.0001**
HOMA-IR	3.45 ± 0.05	2.25 ± 0.03	5.95 ± 0.12	**<.0001**
HbA_1c_, %	5.58 ± 0.01	5.37 ± 0.01	6.02 ± 0.02	**<.0001**
Ca, mg/dL	9.14 ± 0.01	9.11 ± 0.01	9.20 ± 0.01	**<.0001**
Smoking				**<.0001**
No	8524 (54.21%)	5780 (56.56%)	2744 (49.36%)	
Yes	7043 (45.79%)	4349 (43.44%)	2694 (50.64%)	
Drinking				**<.001**
No	2188 (10.92%)	1331 (10.27%)	857 (12.27%)	
Yes	13 379 (89.08%)	8798 (89.73%)	4581 (87.73%)	
Hyperlipidemia				**<.0001**
No	4454 (29.54%)	4076 (40.54%)	378 (6.78%)	
Yes	11 113 (70.46%)	6053 (59.46%)	5060 (93.22%)	
Hypertension				**<.0001**
No	9062 (62.69%)	7357 (76.53%)	1705 (34.06%)	
Yes	6505 (37.31%)	2772 (23.47%)	3733 (65.94%)	
Diabetes				**<.0001**
No	12 534 (85.20%)	9364 (94.97%)	3170 (64.97%)	
Yes	3033 (14.80%)	765( 5.03%)	2268 (35.03%)	

Data are presented as N% (χ^2^ test) or mean ± SD (independent *t* test). Bold font indicates the corresponding *P* values are less than .05, signifying statistical significance.

Abbreviations: ALT, alanine transaminase; AST, aspartate transaminase; BMI, body mass index; Ca, corrected calcium; eGFR, estimated glomerular filtration rate; HbA_1c_, glycated hemoglobin A_1c_; HDL-C, high-density lipoprotein cholesterol; HOMA-IR, homeostatic model assessment for insulin resistance; LDL-c, low-density lipoprotein cholesterol; MDS, magnesium depletion score; TC, total cholesterol; TGs, triglycerides.

### Univariate Logistic Regression for Factors Associated With Metabolic Syndrome

In Table 2, we show the results of univariate logistic regression analysis assessing the association between various factors and MetS. The MDS was found to be significantly associated with increased odds of MetS. The odds ratio (OR) for MDS was 1.78 (95% CI, 1.69-1.88), indicating that every 1-unit increase in MDS was associated with 78% higher odds of having MetS. When MDS was categorized into 6 levels, there was a stepwise increase in the odds of MetS with increasing MDS. Using MDS = 0 as reference, the OR (95% CI) was 1.41 (1.28-1.56) for MDS = 1; 3.12 (2.71-3.59) for MDS = 2; 6.59 (5.28-8.24) for MDS = 3; and 9.05 (5.97-13.73) for MDS = 4. The association was statistically significant for MDS levels ranging from 1 to 4 (all *P* < .0001). Other factors significantly associated with higher odds of MetS included older age, non-Hispanic White race, lower education level, higher BMI, larger waist circumference, elevated levels of ALT, AST, TGs, TC, LDL-c, HOMA-IR, HbA_1c_, smoking, no alcohol drinking, and the presence of hyperlipidemia, hypertension, and diabetes (all *P* < .05).

**Table 2.** Univariate logistics regression analysis of the association between magnesium depletion score and metabolic syndrome

Character	OR (95% CI)	*P*
Age, y	1.04 (1.04-1.04)	**<.0001**
Sex		
Male	Reference	Reference
Female	1.10 (1.00-1.21)	.06
Race		
Non-Hispanic White	Reference	Reference
Non-Hispanic Black	0.80 (0.72-0.89)	**<.0001**
Mexican American	0.90 (0.79-1.03)	.12
Other Race	0.75 (0.65-0.85)	**<.0001**
Education		
> High school	Reference	Reference
High school	1.45 (1.30-1.62)	**<.0001**
< High school	1.67 (1.42-1.97)	**<.0001**
BMI	1.19 (1.17-1.20)	**<.0001**
Waist, cm	1.09 (1.09-1.10)	**<.0001**
MDS	1.78 (1.69-1.88)	**<.0001**
0	Reference	Reference
1	1.41 (1.28-1.56)	**<.0001**
2	3.12 (2.71-3.59)	**<.0001**
3	6.59 (5.28-8.24)	**<.0001**
4	9.05 (5.97-13.73)	**<.0001**
5	1.76 (0.32-9.56)	.51
ALT, U/L	1.02 (1.01-1.02)	**<.0001**
AST, U/L	1.01 (1.00-1.01)	**.002**
TGs, mmol/L	5.42 (4.86-6.04)	**<.0001**
TC, mmol/L	1.07 (1.02-1.12)	**.002**
HDL-c, mmol/L	0.08 (0.07-0.10)	**<.0001**
LDL-c, mmol/L	1.06 (1.01-1.11)	**.02**
HOMA-IR	1.59 (1.53-1.65)	**<.0001**
HbA_1c_, %	3.82 (3.34-4.37)	**<.0001**
Ca, mg/dL	2.29 (1.96-2.68)	**<.0001**
Smoking		
No	Reference	Reference
Yes	1.34 (1.23-1.45)	**<.0001**
Drinking		
No	Reference	Reference
Yes	0.82 (0.73-0.92)	**<.001**
Hyperlipidemia		
No	Reference	Reference
Yes	9.37 (7.80-11.26)	**<.0001**
Hypertension		
No	Reference	Reference
Yes	6.31 (5.65-7.05)	**<.0001**
Diabetes		
No	Reference	Reference
Yes	10.18 (8.92-11.62)	**<.0001**

Bold font indicates the corresponding *P* values are less than .05, signifying statistical significance.

Abbreviations: ALT, alanine transaminase; AST, aspartate transaminase; BMI, body mass index; Ca, corrected calcium; eGFR, estimated glomerular filtration rate; HbA_1c_, glycated hemoglobin A_1c_; HDL-C, high-density lipoprotein cholesterol; HOMA-IR, homeostatic model assessment for insulin resistance; LDL-c, low-density lipoprotein cholesterol; MDS, magnesium depletion score; OR, odds ratio; TC, total cholesterol; TGs, triglycerides.

### Multivariable Logistic Regression Models for the Association Between Magnesium Depletion Score and Metabolic Syndrome

Table 3 presents the results of multivariable logistic regression analysis of the association between MDS and MetS using 4 models. In the crude model without adjustments, a higher MDS was significantly associated with increased odds of MetS. The OR (95% CI) for each 1-unit increase in MDS was 1.78 (1.69-1.88). When MDS was categorized into 6 levels, there was a graded increase in the odds of MetS with increasing MDS (*P* for trend <.0001). After adjusting for demographics (age, sex, race, and education) in model 1, the association between MDS and MetS was attenuated but remained significant. The OR (95% CI) for each 1-unit increase in MDS was 1.33 (1.25-1.41). The odds were also significantly higher for MDS levels 2 to 4 compared to MDS = 0. With further adjustments for various cardiometabolic factors in models 2 and 3, the association between MDS and MetS persisted. In the fully adjusted model 3, the OR (95% CI) for each 1-unit increase in MDS was 1.31 (1.17-1.45). MDS levels 1 to 4 remained significantly associated with higher odds of MetS compared to MDS = 0 after full adjustments (all *P* < .05).

**Table 3.** Multivariable logistics regression analysis of the association between magnesium depletion score and metabolic syndrome

Character	Model							
	Crude model		Model 1		Model 2		Model 3	
	95% CI	*P*	95% CI	*P*	95% CI	*P*	95% CI	*P*
Total	1.78 (1.69-1.88)	**<.0001**	1.33 (1.25-1.41)	**<.0001**	1.51 (1.37-1.67)	**<.0001**	1.31 (1.17-1.45)	**<.0001**
0	Reference		Reference		Reference		Reference	
1	1.41 (1.28-1.56)	**<.0001**	1.00 (0.89-1.11)	.96	1.32 (1.10-1.57)	**.003**	1.28 (1.06-1.55)	**.01**
2	3.12 (2.71-3.59)	**<.0001**	1.60 (1.37-1.87)	**<.0001**	2.10 (1.65-2.68)	**<.0001**	1.57 (1.22-2.03)	**<.001**
3	6.59 (5.28-8.24)	**<.0001**	2.70 (2.09-3.48)	**<.0001**	4.19 (2.78-6.33)	**<.0001**	2.60 (1.69-4.02)	**<.0001**
4	9.05 (5.97-13.73)	**<.0001**	3.49 (2.24-5.44)	**<.0001**	4.76 (2.53-8.94)	**<.0001**	2.83 (1.42-5.61)	**.003**
5	1.76 (0.32-9.56)	.51	0.59 (0.10-3.36)	.55	1.39 (0.12-15.59)	.79	0.88 (0.07-11.47)	.92
*P* for trend		**<.0001**		**<.0001**		**<.0001**		**<.0001**

Crude model: no adjustments made for confounding factors. Model 1: adjustments made for age, sex, race, and education level. Model 2: adjustments made for age, sex, race, education level, BMI, waist, ALT, AST, TGs, TC, HDL-c, LDL-c, HOMA-IR, HbA_1c_, Ca, smoking, and drinking. Model 3: adjustments made for age, sex, race, education level, BMI, waist, ALT, AST, TGs, TC, HDL-c, LDL-c, HOMA-IR, HbA_1c_, Ca, smoking, drinking, hyperlipidemia, diabetes, and hypertension. Bold font indicates the corresponding *P* values are less than .05, signifying statistical significance.

Abbreviations: ALT, alanine transaminase; AST, aspartate transaminase; BMI, body mass index; Ca, corrected calcium; eGFR, estimated glomerular filtration rate; HbA_1c_, glycated hemoglobin A_1c_; HDL-C, high-density lipoprotein cholesterol; HOMA-IR, homeostatic model assessment for insulin resistance; LDL-c, low-density lipoprotein cholesterol; MDS, magnesium depletion score; OR, odds ratio; TC, total cholesterol; TGs, triglycerides.

### Stratified Analysis of the Association Between Magnesium Depletion Score and Metabolic Syndrome

This study examined the association between MDS and MetS across subgroups defined by age, sex, race, BMI, drinking status, and smoking status using multivariable logistic regression analysis adjusted for potential confounders (Table 4). MDS was significantly associated with increased odds of MetS in most subgroups, with ORs ranging from 1.19 to 1.60 per 1-unit MDS increase. The association was not significant only in the Mexican American subgroup (*P* = .08). There was no significant interaction detected between MDS and subgroups, indicating the positive MDS-MetS relationship was consistent across diverse population segments. Overall, these findings demonstrate that higher magnesium deficiency status as reflected by MDS is a robust independent risk factor for MetS irrespective of sociodemographic and lifestyle factors in US adults.

**Table 4.** Subgroup analysis for the association between magnesium depletion score and metabolic syndrome

Character	OR (95% CI)	*P*	*P* for interaction
Age, y			.33
<40	1.26 (1.01-1.58)	**.04**	
40-60	1.28 (1.09-1.51)	**.003**	
>60	1.33 (1.18-1.51)	**<.0001**	
Sex			.46
Male	1.60 (1.40-1.83)	**<.0001**	
Female	1.48 (1.30-1.69)	**<.0001**	
Race			.27
Non-Hispanic White	1.51 (1.34-1.70)	**<.0001**	
Non-Hispanic Black	1.38 (1.22-1.56)	**<.0001**	
Mexican American	1.19 (0.98-1.46)	.08	
Other race	1.37 (1.13-1.65)	**.001**	
BMI			.92
Normal	1.33 (1.02-1.74)	**.03**	
Unnormal	1.50 (1.36-1.64)	**<.0001**	
Drinking			.27
No	1.43 (1.19-1.73)	**<.001**	
Yes	1.50 (1.37-1.64)	**<.0001**	
Smoking			.75
No	1.57 (1.38-1.78)	**<.0001**	
Yes	1.42 (1.26-1.60)	**<.0001**	

Subgroup analysis for the association between MDS and MetS. Weighted univariate logistic regression was used for subgroup analysis. Adjustments were made for education, hyperlipidemia, hypertension, diabetes, waist, ALT, AST, TGs, TC, HDL-c, LDL-c, HOMA-IR, HbA_1c_, and Ca. Bold font indicates the corresponding *P* values are less than .05, signifying statistical significance.

Abbreviations: ALT, alanine transaminase; AST, aspartate transaminase; BMI, body mass index; Ca, corrected calcium; eGFR, estimated glomerular filtration rate; HbA_1c_, glycated hemoglobin A_1c_; HDL-C, high-density lipoprotein cholesterol; HOMA-IR, homeostatic model assessment for insulin resistance; LDL-c, low-density lipoprotein cholesterol; MDS, magnesium depletion score; OR, odds ratio; TC, total cholesterol; TGs, triglycerides.

### Magnesium Depletion Score and Odds of Metabolic Syndrome: A Restricted Cubic Spline Analysis

**Figure 2.**
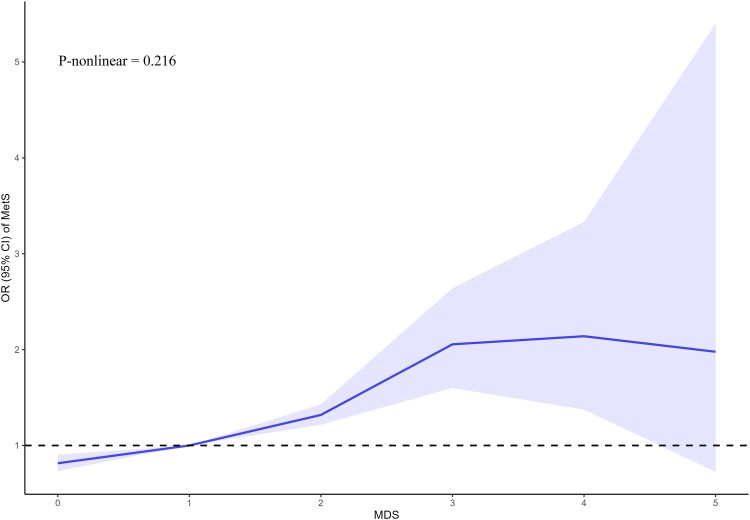
Nonlinear associations between magnesium depletion score (MDS) and risk of metabolic syndrome (MetS). The restricted cubic spline plot between MDS and MetS. The x-axis represents MDS, while the y-axis represents the odds ratio (OR) and the 95% CI for MetS. The dashed line indicates an OR of 1, which represents no association between MDS and MetS risk. The model adjusted by age, sex, race, body mass index, waist, alanine transaminase, aspartate transaminase, triglycerides, total cholesterol, high-density lipoprotein cholesterol, low-density lipoprotein cholesterol, homeostatic model assessment for insulin resistance, glycated hemoglobin A_1c_, corrected calcium, smoking, drinking, hyperlipidemia, diabetes, and hypertension.

As shown in Fig. 2, RCS was drawn to visually describe the relationship between MDS and MetS. The OR increased steadily with higher MDS up to a level of around 4, where the OR peaked. Beyond MDS of 4, the OR plateaued and slightly declined with further increases in MDS. The *P* value for a nonlinear relationship was greater than .05, indicating there was no statistically significant evidence of nonlinearity between MDS and MetS odds. Although the OR appeared to peak at MDS of 4, the overall relationship can be considered linear given the nonsignificant test for nonlinearity. In summary, this figure demonstrates an approximately linear-positive association between higher MDS and increased odds of MetS, without clear threshold effects.

## Discussion

Based on NHANES data from 2003 to 2018, we investigated the relationship between MetS and MDS among US adults. We found that higher MDS, indicative of greater urinary magnesium loss, was independently associated with increased likelihood of MetS. We observed a graded dose-response relationship between MDS and the odds of MetS, with no evidence of a threshold effect. The positive correlation between MDS and MetS remained significant even after adjusting for potential sociodemographic, lifestyle, and cardiometabolic confounders. This association was consistently observed across various subgroups defined by age, sex, race, BMI, drinking status, and smoking status.

MetS is a constellation of multiple risk factors, including insulin resistance, dyslipidemia, hypertension, and adiposity (17). Magnesium is an essential nutrient for maintaining vital physiological functions. Numerous studies have investigated the relationship between MetS and magnesium. An increasing body of evidence suggests that chronic hypomagnesemia may play a role in the pathogenesis of various metabolic disorders, including overweight and obesity, insulin resistance, and type 2 diabetes mellitus, hypertension, alterations in lipid metabolism, and low-grade inflammation (18-20). A randomized, double-blind, placebo-controlled clinical trial show that oral magnesium supplementation improves incident MetS with a significant reduction in high blood pressure, hyperglycemia, and hypertriglyceridemia (21). In conclusion, magnesium plays a substantial role in MetS occurrence and development.

Serum magnesium is the most commonly used approach to evaluate magnesium status in clinical practice. However, it does not accurately reflect the total-body magnesium levels. The serum magnesium level is typically within the normal reference range, especially in the case of chronic magnesium deficiency (22, 23). The serum contains 0.3% of the body's total magnesium, and the rest is primarily stored in bones, muscles, and soft tissue (24). Both urinary magnesium levels and the fractional excretion of magnesium (FEMg) serve as methods for assessing magnesium status. The FEMg is calculated using the concentrations of magnesium and creatinine both in serum and urine, and can serve as a useful tool in assessing magnesium status (25). However, both urinary magnesium and FEMg are easily influenced by various factors such as renal function and dietary intake (26). The magnesium tolerance test is regarded as a gold standard for evaluating the magnesium status of the body, but it is impractical and difficult to apply broadly since it requires a first 24-hour urine collection, intravenous magnesium infusion, and then a second 24-hour urine collection (27, 28). The oral magnesium loading test is another method for assessing magnesium deficiency. This noninvasive procedure merely requires the oral intake of magnesium and urine collection. However, it necessitates the collection of urine over a specific duration, typically 24 hours, which can pose an inconvenience for the patient. This factor may restrict its utilization in routine clinical practice (29). Although intense research activities have been dedicated to magnesium, the difficulties of accessing total-body magnesium, and its main 2 compartments, namely bone and muscle, mean that today there is still no simple, rapid, and accurate laboratory test to indicate total-body magnesium status in humans (28). The MDS has been demonstrated as a good indicator for predicting magnesium deficiency validated by a magnesium tolerance test (12). It is a simple and easy tool to evaluate magnesium deficiency. This implies that the incorporation of MDS with other methods for assessing magnesium status could potentially enhance our ability to effectively identify individuals with magnesium deficiency.

The MDS combines 4 risk factors affecting magnesium reabsorption in the US population, including alcohol consumption, diuretics, PPI, and kidney function (12). For instance, alcohol consumption causes a prompt increase in the urinary excretion of magnesium (30). Similarly, diuretics can enhance magnesium excretion (31). The administration of a PPI has been shown to reduce kidney reabsorption of magnesium by downregulating TRPM6 activity (32). In addition to alcohol and medication use, magnesium reabsorption in the kidney can be drastically altered under certain pathophysiological conditions. Chronic kidney disease is accompanied by increased renal magnesium-wasting (33) and, thus, impaired kidney function has been recognized as an essential pathway underlying magnesium depletion from the urine. Overall, these factors contribute to the utility of MDS as a comprehensive measurement tool for evaluating the status of magnesium.

To our knowledge, there have been no studies investigating the association between magnesium deficiency, assessed using an easy-to-use tool like the MDS, and MetS.

In this study, we discovered a positive association between the MDS, a newly developed assessment metric for magnesium status, and MetS. Our findings are supported by some clinical research. A systematic review and meta-analysis evaluated the theory that a higher magnesium intake is associated with a lower risk of MetS (9 articles, n = 31 876, OR = 0.73; 95% CI, 0.62-0.86; *P* < .001) (34). Other evidence suggests that dietary magnesium has beneficial effects, including regulating systemic inflammation (35) and hypertension (36), regulating lipids (37), glucose and insulin metabolism, improving insulin sensitivity (38), and reducing the risk of diabetes (39). However, data regarding the association between magnesium status and MetS are conflicting. Some studies concluded that serum magnesium levels are lower in patients with MetS compared with their controls (40), whereas other studies reported a nonsignificant association (41) or even proposed that there may be a positive association between serum magnesium concentration and MetS (42). In hypertension, a similar situation exists. A systematic review and meta-analysis of prospective cohort studies found an inverse association between dietary magnesium intake and the risk of hypertension comparing the highest intake group with the lowest. However, the association of serum magnesium concentration with the risk of hypertension was marginally significant (36). This may be because serum magnesium cannot reflect the actual status of whole-body magnesium levels. Therefore, it is very important to find an indicator of the supplement of serum magnesium, to better reflect the status of magnesium levels in the body.

This study sheds light on the issue of magnesium deficiency and uncovers a positive correlation between MDS and the risk of MetS. The results imply that we need to be vigilant about MetS risk, especially for patients with a higher MDS. This allows us to identify individuals in the community who may be in a magnesium-deficient state through the use of MDS and other assessment methods, and provide magnesium supplementation to these populations to reduce the incidence of MetS.

Our study has several advantages. This is the first large, national study to investigate the association between MDS and MetS based on the well-designed NHNAES data. It is worth noting that the analysis incorporates the use of sampling weights assigned to each participant. These weights play a crucial role in enabling statistical inferences and generalizing our findings to a larger population beyond the sample size of MetS cases. By accounting for these weights, our study ensures reliable conclusions and precise statistical power. In addition, a stratified subgroup analysis was conducted to further investigate the relationship between MDS and MetS across different population groups, which suggests that we need to implement more precise prevention strategies for MetS. Furthermore, MDS rather than serum magnesium was used in our study, which is more reflective of the physiological state of magnesium.

This study also has some limitations that need to be clarified. First, we observed that there is a nonsignificant association between MDS and Mets when MDS = 5. This may be attributable to the small number of participants with MDS = 5, which totaled only 6 individuals. Second, we did not evaluate whether MDS was a “better” indicator of magnesium deficiency compared to serum magnesium levels because of the lack of serum magnesium in NHANES. Furthermore, the cross-sectional study design cannot definitively determine causation between MDS and MetS. It is also worth noting that MDS is a categorical variable, not a continuous variable. In addition, while MDS includes PPI and diuretics, other drugs such as tetracyclines, bisphosphonates, β-adrenergic agonists, insulin, etc, which can affect magnesium levels (31), are not accounted for in MDS. Last, although several covariates were adjusted to investigate the association between MDS and MetS, potential confounders that might have effects on the results were not included.

In conclusion, our study identified a significant association between MDS and MetS, and further explored differences across various subgroups. In addition to having major implications for clinical practice, our findings have important public health implications as well. It is possible to prevent and reduce MetS by supplementing with magnesium supplements or encouraging higher magnesium intake diet because diet is a factor that can be changed.

## Data Availability

Publicly available data sets were analyzed in this study. These data can be found at https://www.cdc.gov/nchs/nhanes/index.htm.

## Abbreviations

ALT: alanine transaminase
AST: aspartate transaminase
BMI: body mass index
Ca: corrected calcium
DBP: diastolic blood pressure
eGFR: estimated glomerular filtration rate
FEMg: fractional excretion of magnesium
HbA_1c_: glycated hemoglobin A_1c_
HDL-C: high-density lipoprotein cholesterol
HOMA-IR: homeostatic model assessment for insulin resistance
LDL-c: low-density lipoprotein cholesterol
MDS: magnesium depletion score
MetS: metabolic syndrome
NCEP-ATP III: National Cholesterol Education Program Adult Treatment Panel III Criteria
NHANES: National Health and Nutrition Examination Survey
OR: odds ratio
PPI: proton pump inhibitor
RCS: restricted cubic spline
SBP: systolic blood pressure
TC: total cholesterol
TGs: triglycerides
